# Injury-Induced Innate Immune Response During Segment Regeneration of the Earthworm, *Eisenia andrei*

**DOI:** 10.3390/ijms22052363

**Published:** 2021-02-27

**Authors:** Kornélia Bodó, Zoltán Kellermayer, Zoltán László, Ákos Boros, Bohdana Kokhanyuk, Péter Németh, Péter Engelmann

**Affiliations:** 1 Department of Immunology and Biotechnology, Clinical Center, Medical School, University of Pécs, Szigeti u, 12, 7643 Pécs, Hungary; bodo.kornelia@pte.hu (K.B.); kellermayer.zoltan@pte.hu (Z.K.); kokhanyukb@gmail.com (B.K.); nemeth.peter@pte.hu (P.N.); 2 Department of Medical Microbiology and Immunology, Medical School, University of Pécs, Szigeti u, 12, 7643 Pécs, Hungary; ifj.laszlozoltan@gmail.com (Z.L.); borosakos@gmail.com (Á.B.)

**Keywords:** earthworm, regeneration, innate immunity, coelomocytes, cell proliferation, apoptosis, gene expression

## Abstract

Regeneration of body parts and their interaction with the immune response is a poorly understood aspect of earthworm biology. Consequently, we aimed to study the mechanisms of innate immunity during regeneration in *Eisenia andrei* earthworms. In the course of anterior and posterior regeneration, we documented the kinetical aspects of segment restoration by histochemistry. Cell proliferation peaked at two weeks and remitted by four weeks in regenerating earthworms. Apoptotic cells were present throughout the cell renewal period. Distinct immune cell (e.g., coelomocyte) subsets were accumulated in the newly-formed blastema in the close proximity of the apoptotic area. Regenerating earthworms have decreased pattern recognition receptors (PRRs) (e.g., *TLR,* except for *scavenger receptor*) and antimicrobial peptides (AMPs) (e.g., *lysenin*) mRNA patterns compared to intact earthworms. In contrast, at the protein level, mirroring regulation of lysenins became evident. Experimental coelomocyte depletion caused significantly impaired cell divisions and blastema formation during anterior and posterior regeneration. These obtained novel data allow us to gain insight into the intricate interactions of regeneration and invertebrate innate immunity.

## 1. Introduction

The regeneration capacity is a poorly understood aspect of biology and explaining its variation among animals remains elusive [1]. Great efforts have been made to elucidate the ecological perspectives, phylogenetic distribution and developmental patterns of restoration mechanisms. The extensive regeneration ability initially flourished at the dawn of animal evolution presumably coinciding with the origin of multicellularity; however, limitations or loss of regeneration capability have been widespread across the phylogenesis [2]. Among vertebrates, the directionality of the lost regeneration machinery is less obvious. It is hypothesized that in higher developed vertebrates’ the regeneration of whole organs and body parts is hampered by the evolution of the adaptive immune system that determines only wound-healing and scarring following the tissue injury. In this light, the transition into warm-blooded vertebrates required prompt responses instead of whole-body part or organ regeneration [2,3,4,5,6,7].

Among invertebrates, annelids operating only with innate immunity have extensive regeneration capacity [8,9]. The restoration process in earthworms depends on food availability, developmental stages, and the exact position and the number of lost segments [9,10]. During evolution, the regeneration ability of earthworms has evolved probably due to predatory pressure and is achieved by the combination of epimorphic recovery and morphallactic transformation of old segments. Epimorphosis concomitantly results in an embryonic-like tissue formation, the so-called blastema (a cluster of undifferentiated cells) [2,5,10,11]. Regeneration is well-studied morphologically, but the molecular background in *Oligochaeta* earthworms is currently less understood. Such studies have focused on specific genes that also have a crucial role in the development of *Platynereis* and *Pristina* earthworms (e.g., *hox* and *hedgehog* genes) [12,13].

Invertebrates have been long exerted to investigate the evolution of immunity [14]. Earthworms possess refined and efficient innate immune mechanisms against environmental pathogens. During animal development, the close interactions between cellular and humoral immune compartments became apparent here first [15,16]. Earthworm’s coelomocytes (macrophage-like cells) are part of the cellular immune response, which are both morphologically and functionally analogous to vertebrate phagocytes [16]. Coelomocyte subpopulations (named as hyaline-, granular amoebocytes and eleocytes) possess distinct functions such as phagocytosis, encapsulation and cellular cytotoxicity [15,17]. Relying on our previous findings, hyaline and granular amoebocytes are capable of phagocytosis and encapsulation [16]. Their pathogen recognition is accomplished by an assortment of pattern recognition receptors (PRRs) and their distinct expression was also proven at the mRNA level [18,19]. Eleocytes play a detoxifying role through their lysosomal system and more importantly, they produce diverse bioactive proteins (e.g., lysenins) that participate in the humoral immune responses [15,16].

Until now, little is known about interactions between regeneration and immune mechanisms in the different animal groups (not only in earthworms but also in higher vertebrates). Studies have shed light on the pivotal role of the immune system in efficient regeneration [3,6]. Evidence suggests that the regenerative capacity is inversely proportional with the activity of the immune system of vertebrates [6]; however novel observations claim the promotion of regeneration with the functional immune system [7].

Molecular aspects of the segment restoration (with an exception of some genes that can be linked to the developmental process) [20] are largely unexplored. In this study, we approach specifically the regeneration process from the perspective of the immune response. Our principal aims were to determine cell proliferation and cell death in relation to coelomocytes during rostral and caudal segment restoration of the earthworm, *Eisenia andrei*. Therefore, in parallel with morphological/cytological observations, we monitored the expression of certain immune-related gene families (pattern recognition receptors and antimicrobial molecules) in the course of anterior and posterior regeneration in *E. andrei*. Finally, we scrutinized that the coelomocyte ablation impaired the normal circle of the regeneration process in earthworms.

Our novel discoveries might provide further mechanistic insights into how the innate immune response is involved in the tissue repair of earthworms.

## 2. Results and Discussion

### 2.1. Morphological Changes During Anterior and Posterior Earthworm Regeneration

**Figure 1:**
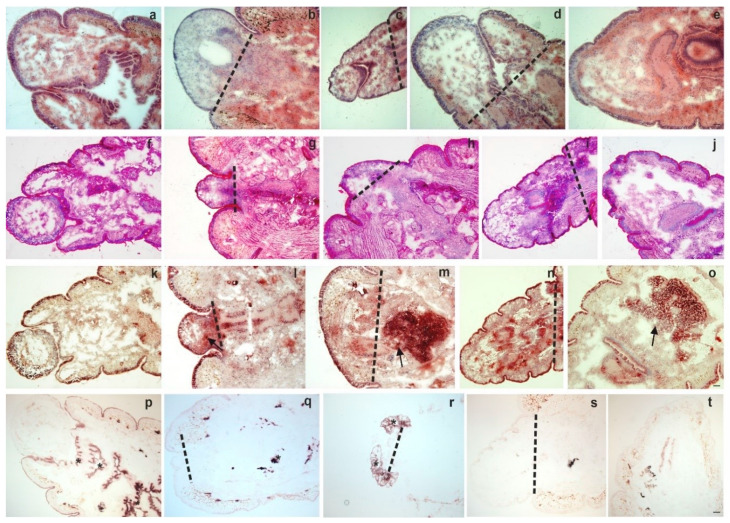
Histochemistry of intact anterior segments (**a**,**f**,**k**,**p**) and regenerating blastema at various time points: 4 days (**b**,**g**,**l**,**q**); 1 week (**c**,**h**,**m**,**r)**; 2 weeks (**d**,**i**,**n**,**s**) 4 weeks (**e**,**j**,**o**,**t**). Haematoxylin-eosin (H&E) (**a**–**e**), Periodic acid and Schiff reaction (PAS) (**f**–**j**), acid phosphatase (ACP) (**k**–**o**) and alkaline phosphatase (ALP) (**p**–**t**) stainings were performed. Representative images were presented from five independent experiments. Arrows point to ACP (**m**–**o**) positive cells. Asterisks mark ALP (**q**–**r**) expressing cells. The level of amputation is indicated by a dashed line. Scale bars: 200 μm.

**Figure 2:**
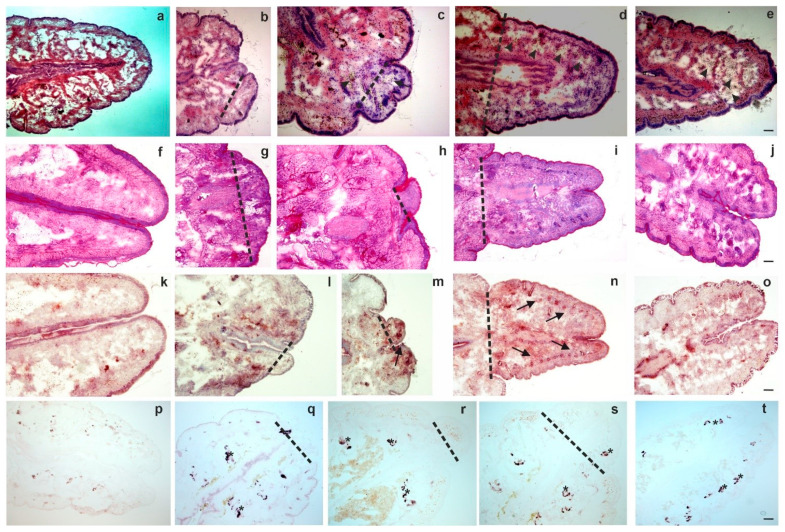
Histochemistry of intact posterior segments (**a**,**f**,**k**,**p**) and regenerating blastema at various time points such as 4 days (**b**,**g**,**l**,**q**); 1 week (**c**,**h**,**m**,**r)**; 2 weeks (**d**,**i**,**n**,**s)**; 4 weeks (**e**,**j**,**o**,**t**). Standard histochemical staining methods were performed: (**a**–**e**) H&E, (**f**–**j**) PAS, (**k**–**o**) ACP and (**p**–**t**) ALP. In H&E images arrowheads point to coelomocytes (**c**–**e**) in the blastema. Representative images were selected from five independent experiments. Arrows point to ACP (**l**–**n**) expressing cells. Asterisks represent cells with ALP (**q**–**t**) expression. The level of amputation is denoted by a dashed line. Scale bars: 200 μm.

Earthworm regeneration has been well-studied morphologically, however, the underlying molecular and immunological background is largely unknown. Their segment restoration is mediated by epimorphosis, which results in a blastema (an unpigmented mass of undifferentiated cells) formation [5,11]. Following the amputation of the first or last five segments, we observed the stages of typical annelid regeneration in *E. andrei* earthworm within four weeks (Figure 1 and Figure 2). Briefly, the wound of the severed segments immediately closes, shortly heals and later epiblasts (undifferentiated cells) cover it and extend on its surface. Actively proliferating cells in the vicinity of the wound create the blastema constituted by morphologically distinct cell types [8]. According to Bely and Sykes [8], myoblasts can be found underneath the proliferation zone, most likely originating from the body wall and intestinal tract; endoblasts and mesodermal cells with migratory ability can also be seen in the lower layers. The blastema constantly grows and eventually differentiates to substitute structures of which animals have been deprived [11].

Employing general and enzyme histochemical staining methods (for methodology please see the Supporting Material) first we observed the kinetics of anterior (Figure 1a–t) and posterior (Figure 2a–t) regeneration. We observed the blastema from its clear appearance (day 4) until the end of the regeneration period (four weeks).

Formation of new segments and tissue reorganization was documented during anterior (Figure 1a–e) and posterior (Figure 2a–e) regeneration. PAS staining showed no considerable differences between intact and anterior (Figure 1f–j) or posterior (Figure 2f–j) regenerating animals. In contrast, targeting acid phosphatase (ACP) activity, substantial alterations between intact and regenerating earthworms were observed (Figure 1k–o; Figure 2k–o). This lysosomal enzyme had increased activity in the cells of the anterior and posterior blastema. The most pronounced activity was observed in the course of one weekk and two weeks of restoration, which can be a sign of increased activity of phagocytes [15] or a marker for elevated autophagy during regeneration [21].

Alkaline phosphatase (ALP) activity was present in mesodermal tissues of both intact and regenerating earthworms. In the course of anterior regeneration (Figure 1p–t) ALP-positive cells were markedly elevated in early blastema formation (four days and one week regenerating earthworms). In contrast, ALP positive cells could be observed throughout the posterior restoration (Figure 2p–t). Cells with ALP activity occupied a more posterior position mainly running along the septum and intestinal tract as well as in muscle layers (Figure 2p–t). ALP possesses a variety of functions in many animal taxa throughout evolution and its role can also be related to regeneration as well as stem cell activity [22,23].

### 2.2. Cell Proliferation and Tissue Remodelling in the Regenerating Blastema

**Figure 3:**
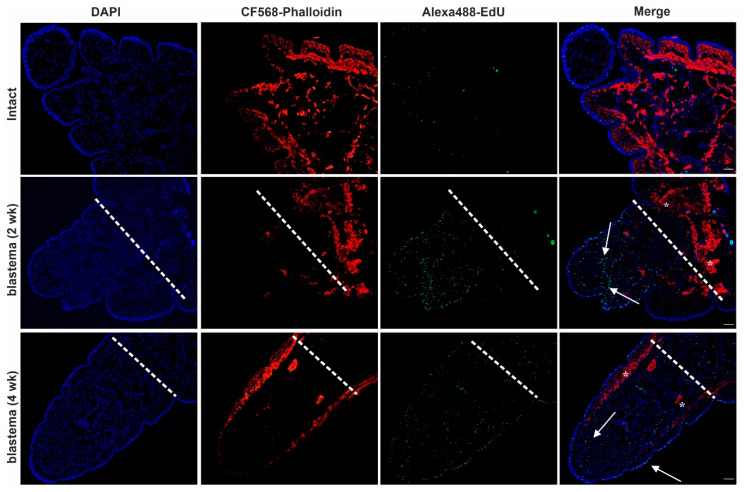
Cell division and tissue re-organization in intact earthworms and in 2- and 4-week anterior blastema. The blastema is marked by a dashed line. Representative images were chosen from five independent experiments. Arrows point to the EdU-positive cells (green). Asterisks mark the reorganized actin filaments (red) in the blastema. The level of amputation is indicated by a dashed line Scale bars: 200 μm.

**Figure 4:**
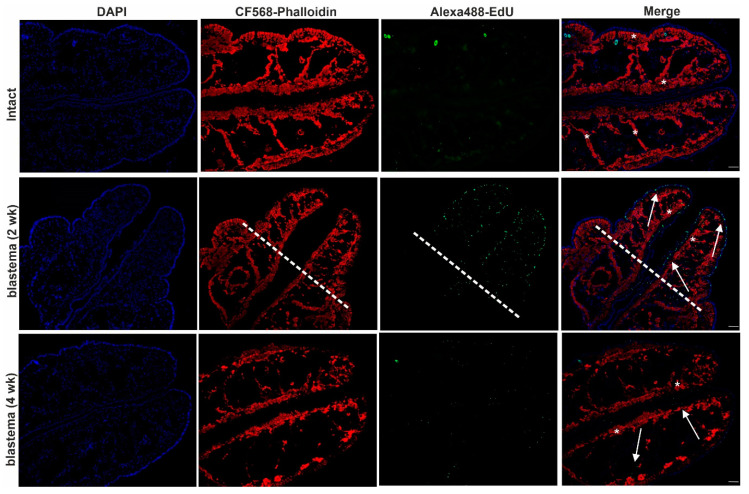
Cell division and tissue re-organization in intact earthworms and in 2- and 4-week posterior blastema of regenerating earthworms. Representative images were chosen from five independent experiments. Arrows point to EdU-positive cells (green) and asterisks remark the actin filaments (red) in the blastema. The level of amputation is denoted by a dashed line. Scale bars: 200 μm.

To detect cell proliferation, we applied EdU-staining in intact, two- and four-week-old regenerating animals (time points were chosen based on the most noticeable morphological differences). We combined the EdU-staining with phalloidin labeling to confirm the rearrangements of actin filaments throughout the regeneration period. A high number of dividing cells were detected in the anterior blastema after two weeks, which barely diminished by week 4 compared to intact earthworms (Figure 3). Caudal regeneration was also completed by week 4, similarly to the anterior counterpart (Figure 4).

Furthermore, compared to intact animals, cell proliferation was more obvious after two weeks in the posterior blastema that predominantly remitted by week 4 (Figure 4) (assessment of dividing cells throughout restoration is detailed in 2.7). EdU-staining was initially combined with phalloidin labeling to ascertain the actin cytoskeletal rearrangements possibly liable for morphological alterations cognate with cell motility and phagocytic capability. Differential actin organization in intact and regenerating earthworms (Figure 3 and Figure 4) yielded evidence that actin is dynamically reorganized and appears as an evolving network during the regeneration period. The continuous formation of new fibers during anterior regeneration was apparent, which resulted in more explicated actin tracking in the four-week-old anterior blastema (Figure 3). By contrast, there were fewer differences between posterior intact segments and regenerating blastema (Figure 4).

We also monitored the tissue reorganization targeting specifically the smooth muscle α actin (α-SMA) by immunofluorescence (please see Supplementary Material: Figure S1a,b). In higher developed vertebrates the functions of α-SMA are extensively studied. Its expression is overwhelmingly associated with smooth muscle and other specialized cells, including myofibroblasts (the most reliable marker to define their phenotype), which own a characteristics role in tissue repair [24,25].

We observed that α-SMA was not present in two-week anterior blastema, but it only appeared in the original segments (Figure S1a). Interestingly, during week 2 of caudal regeneration α-SMA was barely visible in the blastema (Figure S1b) which was in stark contrast to the correspondent phalloidin staining (Figure 4). By four weeks of regeneration, α-SMA was strongly expressed in the muscle layers of both anterior and posterior blastema. Our data raise the possibility regarding the evolutionarily conserved role of α-SMA since its continuous de novo expression or activation in newly-formed segments was the most striking observation in the regenerating blastema.

### 2.3. Coelomocytes are Accumulated in the Regenerating Blastema

The cellular and humoral components of innate immunity play a pivotal role in regeneration and additionally, their activity determines its successful completion [26]. Coinciding with this ascertainment, it is likely that earthworm coelomocytes are involved in the progress of segment restoration [27]. Therefore, we wished to unravel the immunobiological processes during anterior and posterior regeneration of *E. andrei* earthworms.

**Figure 5:**
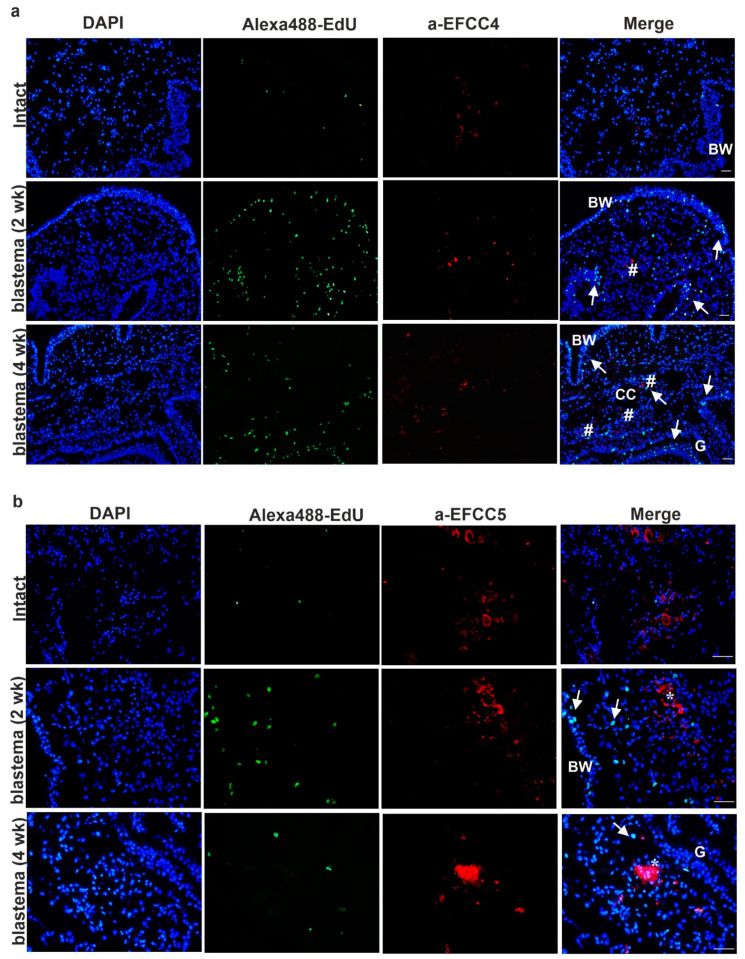
Detection of cell division and localization of coelomocytes by specific mAbs in intact earthworms and 2- and 4-week anterior blastema: (**a**) granular amoebocytes (EFCC4-positive cells) and (**b**) eleocytes (EFCC5-positive cells). Arrows point to proliferating EdU-positive cells (green). (**a**) Number signs mark granular amoebocytes (red) and (**b**) asterisks denote eleocytes (red). Representative images were selected from five independent experiments. BW—body wall; CC—coelomic cavity; G—gut. Scale bars: 100 μm.

**Figure 6:**
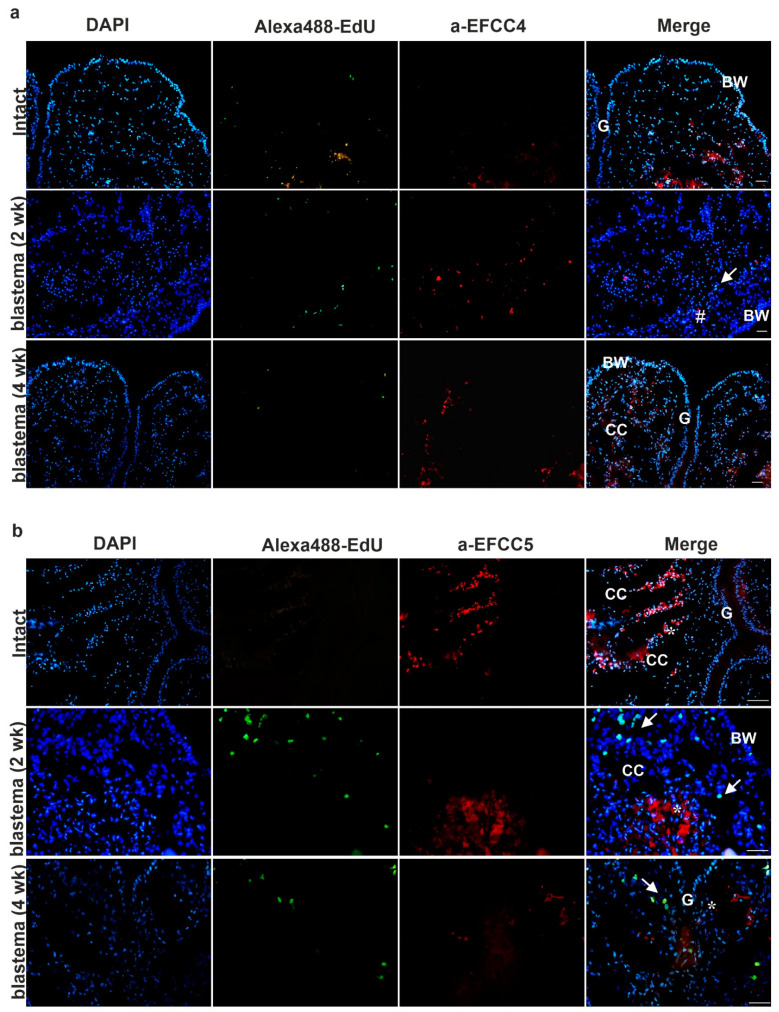
Detection of cell proliferation and localization of coelomocytes by specific mAbs in intact segments, and 2- and 4-week posterior blastema: (**a**) granular amoebocytes (EFCC4-positive cells) and (**b**) eleocytes (EFCC5-positive cells). Arrows point to dividing EdU-positive cells (green). (**a**) Number signs mark granular amoebocytes (red) and (**b**) asterisks indicate eleocytes (red). Representative images were selected from five independent experiments. BW—body wall; CC—coelomic cavity; G—gut. Scale bars: 100 μm.

To this end, subsequently, to EdU based detection of cell-replacement, we applied anti-EFCC4 (granular amoebocyte-specific) and anti-EFCC5 (raised against lysenin protein that is mostly produced by the eleocyte subgroup) monoclonal antibodies (mAbs) that were previously developed against *Eisenia* coelomocytes [15,17]. With the help of these coelomocyte-specific mAbs, we were able to confirm the participation of distinct coelomocyte subsets in the regeneration process. Granular amoebocytes and free-floating eleocytes migrated to the newly growing (regenerating) anterior (Figure 5a,b) and posterior (Figure 6a,b) segments.

However, fewer EFCC4-positive granular amoebocytes and EFCC5-positive eleocytes were observed in the anterior regenerating segments (Figure 5a,b) compared to the posterior counterparts (Figure 6a,b). In contrast, localization of EFCC5 positive eleocytes was apparent and eleocytes formed clusters in the lacunae of the two-week posterior blastema (Figure 6b), although they showed a more diffuse distribution in intact animals (Figure 5b and Figure 6b). The presence of lysenin-secreting eleocytes (EFCC5) probably creates an antimicrobial environment that is more favorable during the regeneration process. Moreover, eleocytes can supply the growing tissues with certain nutrition factors [15,16].

EFCC4-positive granular amoebocytes were also present in the regenerative blastema, whereas, not only in coelomic cavities but also in the body wall, alimentary canal and muscle layers (Figure 5a and Figure 6a). These phagocytic coelomocytes migrate to the proximity of the lesion and probably eliminate the damaged epithelial and muscle cells (Figure 5a and Figure 6a) [28].

Our findings are further supported by the active involvement of immune cells in the regeneration of the central nervous system (CNS), demonstrated in a crustacean (*Ucides cordatus*) [29]. Furthermore, several antimicrobial peptides (AMPs) (e.g., theromacin, neuromacin, *Hm*-lumbricin) have been shown to enhance the repair of damaged axons in the CNS of the leech *Hirudo medicinalis* [30].

### 2.4. Programmed Cell Death and Coelomocytes in the Course of Regeneration

**Figure 7:**
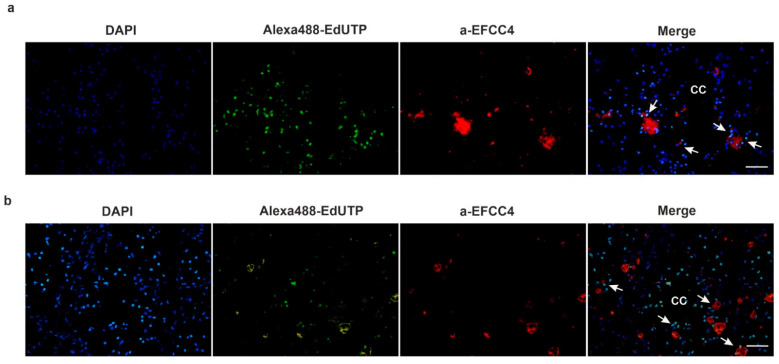
Detection of apoptosis and localization of EFCC4-positive granular amoebocytes in the 4-week anterior (**a**) and posterior (**b**) blastema. The TUNEL positive cells were overwhelmingly increased after 4 weeks and granular amoebocytes (red) were also present in the close vicinity of apoptotic cells (green). CC-coelomic cavity. Arrows point to TUNEL positive cells. Representative images were presented from five independent experiments. Scale bars: 100 μm.

In parallel with cell proliferation, programmed cell death (PCD, apoptosis) is indispensable to successful regeneration [30]. By applying the Click-iT TUNEL assay we unraveled that apoptosis was present during the entire regeneration process, however, it showed a certain fluctuation. The first intensive apoptotic sign was noticed during the wound-healing, but its diminution was observed in two-week regenerating animals (Figures S2 and S3). A burst of cell death was detected in four-week anterior (Figure 7a) and posterior (Figure 7b) regenerating blastema.

This observation suggests that apoptosis was particularly elevated in early blastema formation and during the trans-differentiation phase. During the regeneration of *Hydra* (head and feet restoration take about three days), apoptosis similarly occurred in two apoptotic peaks. The first wave occurred within an hour of the intervention, while the other considerably later [31]. These dying cells release Wnt3, which triggers the activation of the Wnt/β-catenin pathway that is a critical part of *Hydra*’s head regeneration. Thus, these apoptotic cells initiate cell proliferation and can open paths to regeneration [31,32]. In the *Schmidtea mediterranea* planaria, the first apoptotic wave could be tied to wound-healing, although the second was specific to regeneration [33]. In irradiated *Drosophila* larvae, ~60% of the wing imaginal disc cells were able to reconstruct to the normal size through apoptosis-induced cell proliferation [32,34]. Two massive apoptotic waves were also observed during the caudal fin regeneration of adult zebrafish [31,35]**.**

Earthworm amoebocytes are capable of migration and phagocytosis, therefore they are most probably involved in the clearance mechanism and tissue remodeling during regeneration. We found that EFCC4-positive granular amoebocytes were localized in the close vicinity of apoptotic cells (Figure 7a,b). They were also noticeable in the newly formed blastema, the coelomic cavity and muscle layers. In vertebrates, assorted cell types of innate immunity (neutrophil granulocytes, monocytes, macrophages, etc.) migrate to the site of injury and are engaged in the removal of cellular debris [36]. Their protection against pathogens contributes to efficient wound-healing and recovery. During zebrafish caudal renewal the role of macrophages is thoroughly investigated and is fundamental for successful tissue reorganization [37]. According to our results, earthworm phagocytes are also involved in segment restoration. It has been noted that the second apoptotic wave enables the rearrangement of existing structures, and the presence of coelomocytes influences the success of blastema formation [38,39].

### 2.5. Biased Immune-Related Gene Expression during Anterior and Posterior Regeneration

Until recently, most regenerative studies (including earthworms and higher developed organisms) have focused almost exclusively on specific gene expression involved in development. In the early stages of regeneration, the blastema becomes transcriptionally active. Variations in the expression of several *Hox* cluster genes, *GMP* genes, *Wnt* signaling pathway genes, *glutamine synthetase*, *twist*, *miRNAs*, *grimp* and *tuba*, etc. were documented during annelid regeneration [8,40,41,42,43,44,45,46,47,48,49,50].

**Figure 8:**
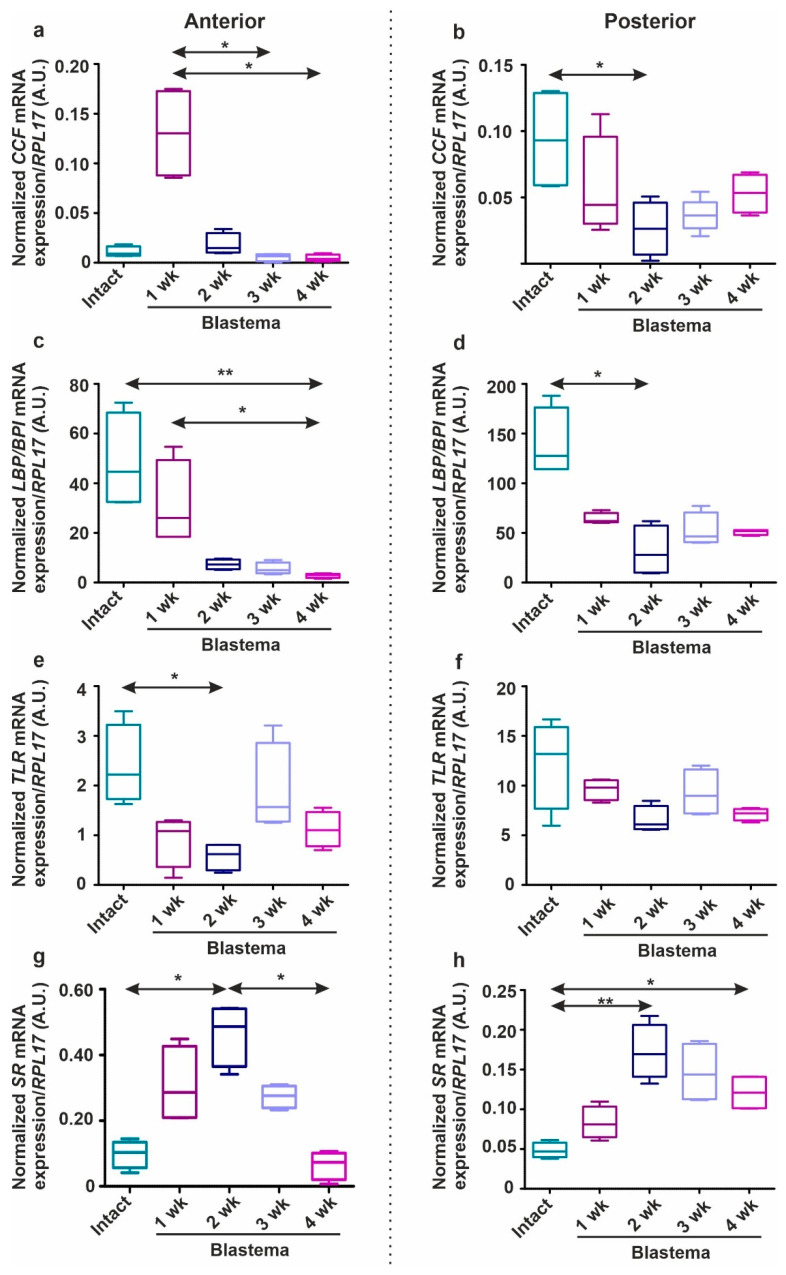
Expression patterns of PRR genes (*CCF*: (**a**,**b**)*; LBP/BPI*: (**c**,**d**)*; TLR*: (**e**,**f**)*; SR*: (**g**,**h**)) during the anterior (**a**,**c**,**e**,**g**) and posterior (**b**,**d**,**f**,**h**) regeneration from 1 to 4 weeks. Gene expressions in the regenerating blastema were compared to intact segments. The boxes represent the interquartile ranges (IQR), whiskers denote lowest and highest values, horizontal lines indicate the median of four independent (*n* = 4) experiments that were carried out in duplicates. The significance of the data was evaluated by one-way ANOVA with Dunnett’s post-hoc test using GraphPad Prism software (* *p* < 0.05, ** *p* < 0.01). Results were normalized to *RPL17* mRNA level. A.U. arbitrary unit.

**Figure 9:**
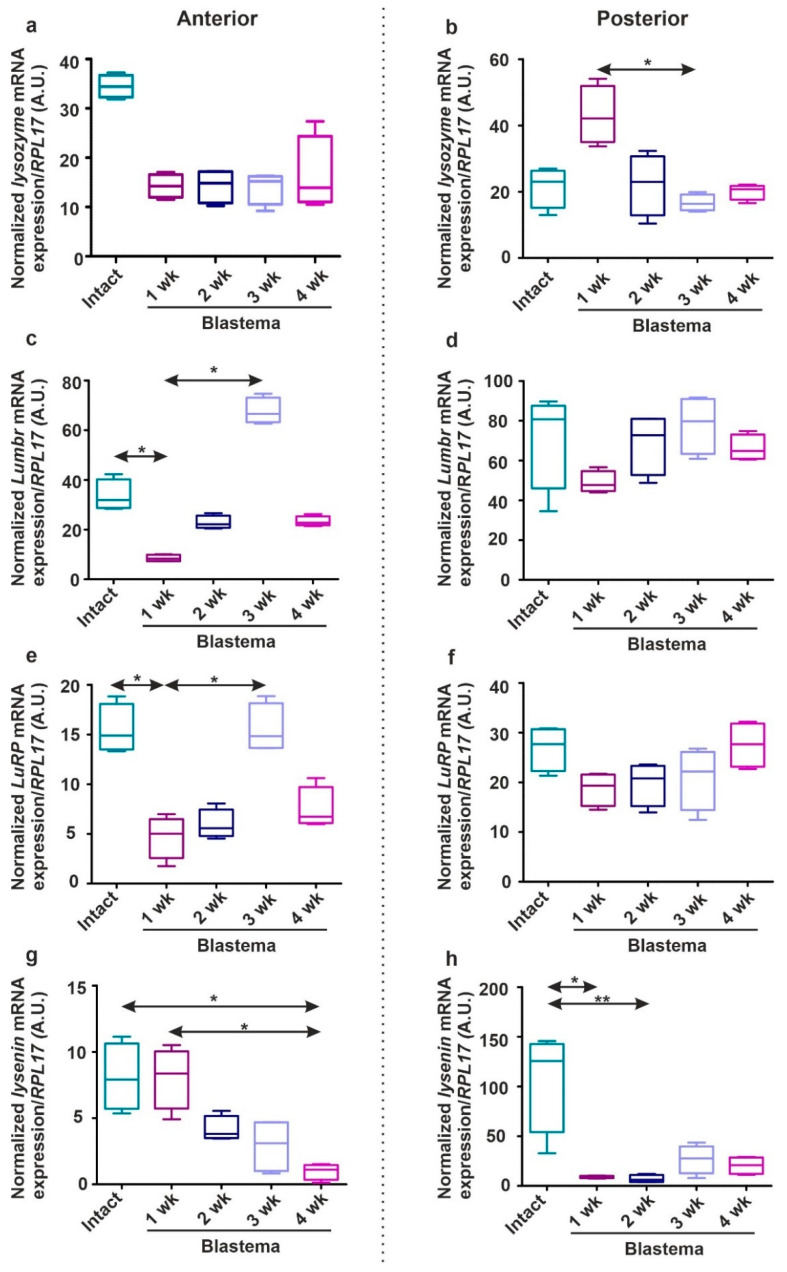
Expression patterns of AMP genes (*lysozyme*: (**a**,**b**); *Lumbr*: (**c**,**d**); *LuRP*: (**e**,**f**); *lysenin*: (**g**,**h**)) during anterior (**a**,**c**,**e**,**g**) and posterior (**b**,**d**,**f**,**h**) restoration from 1 to 4 weeks. Gene expressions of regenerating blastema were compared to intact ends. The boxes represent interquartile ranges (IQR), whiskers signify lowest and highest values, horizontal lines designate the median of four independent (*n* = 4) trials that were executed in duplicates. The significance of the data was estimated by one-way ANOVA with Dunnett’s post-hoc test with GraphPad Prism software (* *p* < 0.05, ** *p* < 0.01). *RPL17* mRNA level used for normalization. A.U. arbitrary unit.

During earthworm regeneration, immune response-related gene expressions have not been scrutinized yet. In our experiments, we followed the kinetical changes in mRNA patterns of PRRs (*TLR*, *CCF*, *LBP/BPI*, *SR*) (Figure 8) and AMPs (*lysenin*, *lumbricin*, and *lumbricin-related peptide*) (Figure 9) throughout anterior and posterior regeneration (reagents and methodology (applied primer sequences are listed in Table S1) are detailed in the Supplementary Materials).

CCF is an earthworm-specific PRR that recognizes a broad range of pathogen structures (the O-antigen of LPS, β-1,3-glucans, and peptidoglycan) [19]. During early anterior regeneration, an increased mRNA expression of *CCF* was found in the blastema (Figure 8a). Contrastingly, in the course of the posterior counterpart, its expression was diminished at two weeks, but it became slightly elevated towards four weeks (Figure 8b). LBP/BPI molecules exist both in invertebrates and vertebrates. In comparison with other homologues, *E. andrei* LBP/BPI was also presumably involved in the binding of LPS [51]. *LBP/BPI* demonstrated rather similar, but lower expression levels throughout the anterior (Figure 8c) and posterior (Figure 8d) segment restoration.

TLR and scavenger receptor (SR) homologs have been found from sponges to mammals [52,53]. *E. andrei TLR* showed elevated mRNA expression upon Gram-negative bacterial stimulation [54]. The down-stream events of TLR ligand-binding in earthworms were not identified yet, however, in vertebrates, TLR activation leads to the synthesis of inflammatory cytokines (e.g., TNFα and INFγ) [55]. In insects, Toll ligand provoked the production of AMPs [56].

In anterior and posterior blastema, *TLR* evidenced similar levels over time, however, its expression seemed to be decreased in contrast to intact segments (Figure 8e,f).

In mammals, SRs bind and internalize a variety of microbial pathogens as well as endogenous ligands (e.g., apoptotic cells), which contributes to a range of altered physiological and pathological modifications [57]. To date, no detailed information was revealed about the exact role of SRs in earthworms. Interestingly, during anterior (Figure 8g) and posterior restoration (Figure 8h) we observed the transient augmentation of *SR* mRNA level towards week 2 that was decreased by week 4. This phenomenon precedes the increase of apoptotic cells (Figure 7) in the regenerating blastema.

To understand any connections between the expressions of earthworm PRRs and antimicrobial factors during regeneration, we investigated the *AMP* levels in the course of segment restoration. Regenerating *E. andrei* revealed markedly lower expression of evolutionarily conserved *lysozyme* compared to intact earthworms (Figure 9a,b), however, one-week posterior blastema (Figure 9b) showed an elevated *lysozyme* mRNA level. Recently, we identified and characterized the proline-rich lumbricin and its close-relative molecule (LuRP) in *E. andrei* earthworms [58]. The medicinal leech microbial challenge enhanced *lumbricin* and *neuromacin* expression, which most probably promoted neuronal regeneration [30]. Both *Lumbr* (Figure 9c,d) and *LuRP* (Figure 9e,f) AMPs demonstrated largely attenuated mRNA expression (except a partial elevation at three weeks of anterior blastema) during the regeneration period compared to intact animals. (Figure 9c–e and Figure 9d–f).

*Eisenia*-specific lysenins are multitasking (cytotoxic, antibacterial, etc.) molecules produced mainly by eleocytes [59]. During regeneration, *lysenin* expression was remitted most remarkably compared to other tested genes. Its persistently decreased expression was the major characteristic of the observed regeneration period (Figure 9g,h) compared to other AMPs (e.g., *Lumbr* and *LuRP*).

Pathogens and “danger signals” (e.g., heat shock proteins, extracellular ATP, IL-1α, IL-33) are recognized by TLRs and other PRRs in injured mammals. This initiates inflammatory processes through the activation of NF-κB. Otherwise, this protective immune response is an inescapable entailment of tissue damage and uncontrolled inflammation negatively regulates the tissue renewal after injuries and delays the healing mechanisms [3,4,55,60]. However, following tissue damages, the inflammatory response is a normal process and facilitates the repair by recruiting innate immune cells [4]. Previously in *Hydra*, this statement was also fortified at the transcript level, where the immediate and early immune responses were monitored followed by bisection [61]. After the mid-gastric bisection, 43 evolutionarily conserved genes were transiently upregulated during the first hours in either head and foot regeneration tips of *Hydra*. Furthermore, the immediate production of H_2_O_2_ in the vicinity of the wound has resulted in the concomitant activation of stress-related ROS target and MAPK pathway genes. To conclude these outcomes the instant activation of ROS signaling genes was in tight connection with several immune genes, which were also upregulated in the proximity of the bisection site. These multiple early regulations propose that elevated expression of genes was rather associated with wound-healing than to a specific regeneration program. Otherwise, key elements of the *Hydra* innate immunity (e.g., NF-κB, TLR, caspases, etc.) were not modulated at the genetic level, therefore these aspects of their regeneration require further investigations [61]. In planaria (*S. mediterranea*), many homolog genes of the innate immunity were triggered upon injury and activated during the restoration program untangled by comparative genome analysis. The transcriptome analysis evidenced diverse expression of immune-related genes throughout the early and later regeneration period, however, coinciding with our data the downregulation of *TIR* expression was documented during the later stages of regeneration (after 24–72 h of amputation) [62]. Remarkably, more empirical data are available from vertebrates regarding the connection of the immune system and tissue restoration mechanisms. A recent molecular analysis revealed a strong down-regulation of immune genes in the regenerating tail of the wall lizard (*Podarcis muralis*), while the Wnt-pathway and non-coding RNAs were upregulated. Conversely, a strong inflammation was experienced in the regenerating limb by upregulation of inflammatory immune-genes along with the simultaneous appearance of Wnt-repressors and the obvious absence of non-coding RNAs. It is likely that downregulation of immune genes in the tail accompanied with upregulation of members of Wnt-pathway and non-coding RNAs prepare the blastema to an immunologically tolerated embryonic-like tissue [63]. A comprehensive analysis in the axolotl (*Ambystoma mexicanum*) proved that inflammation influences the initiation and completion of limb regeneration. These results shed light on the rapid induction of cytokines, chemokines, and inflammatory markers within one day of limb amputation. Surprisingly, early upregulation of anti-inflammatory cytokines was also described; consequently, the growing blastema may recapitulate the embryonal developmental program which is indispensable for directing restoration [64]. In the developing anuran *Xenopus laevis* limb regeneration, an inverse correlation between inflammatory response and regeneration capacity further underscored that the blastema is an immune-privileged organ [65]. Relying on one hypothesis in higher vertebrates the process of effective regeneration is impeded by the development of the adaptive immune system accompanied by a newly organized signaling system that provokes only wound-healing instead of whole-body parts regeneration [3,63].

Our chronological results at the molecular level designate the dysregulation of the studied genes that may indicate the immature feature of the blastema-forming cells and its metabolism may also deviate from the differentiated tissues. According to another theory, down-regulation of immune response genes is likely to be explicable with distinct allocation strategies and life-history “trade-offs” between regeneration and immune functions [2]. In this light, both regeneration and immunity are costly in terms of energy and nutrient usage. Organisms distribute their constrained resources between the competing activities probably leading to trade-offs. The primary goal during earthworm regeneration is to promptly replace lost segments. Therefore, in the sense of trade-off during the time of restoration, much of the absorbed energy is devoted to the regeneration mechanism, and thus probably less energy is allocated to the defense mechanisms [66]. Further observations will be necessary to distinguish this proposal from other feasible explanations for better comprehension.

### 2.6. Lysenin Proteins are Inversely Secreted During Anterior vs. Posterior Regeneration

Most regenerative biological studies were focused on the molecular patterns of different genes [20,63]. Fundamental questions regarding protein expression levels are largely neglected. Members of the lysenin protein family (e.g., lysenin and lysenin-related proteins) are considerable components of the coelomic fluid and have a variety of functions [16,59,67]. Severed earthworms are more susceptible to infections caused by soil pathogens; subsequently, antimicrobial proteins are essential during the regeneration to defend the organisms against infectious agents.

**Figure 10:**
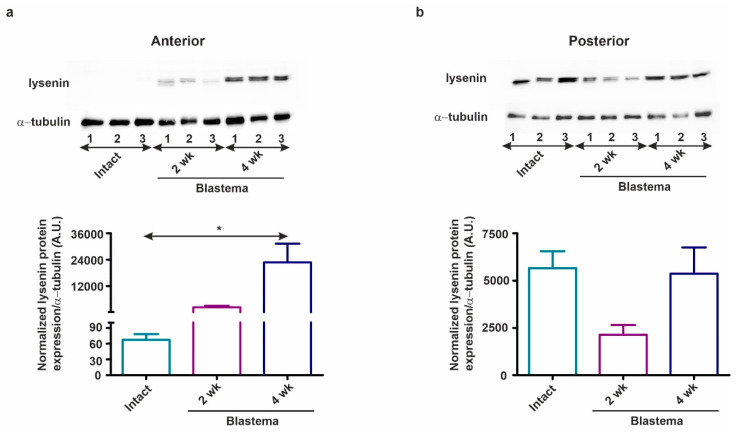
The protein profile of lysenins was studied by Western-blot during (**a**) anterior and (**b**) posterior restoration in intact earthworms and in 2 and 4 weeks regenerating blastema. Upper bands supposedly correspond to lysenin-related protein 2 (~40 kDa) and lower bands to lysenin (~37–38 kDa). Three independent experiments are presented (*n* = 3). The graphs below indicate the band intensity normalized to the corresponding protein band of α-tubulin as reference protein. The values illustrate mean ± SEM.

This assumption was corroborated by the data obtained from our Western blot studies. During anterior and posterior regeneration lysenins have differential expressions in the corresponding blastema (Figure 10a,b) (Technical details are presented in the Supplementary Material). In the rostral blastema, lysenin was significantly increased by week 4 of restoration compared to intact animals (Figure 10a). Meanwhile, during week 2, caudal regeneration lysenin expression was decreased, but by week 4 of regeneration, its expression level was restored to those of intact tissues (Figure 10a). This protein expression pattern is opposed to the observation of *lysenin* mRNA level (Figure 9g,h). This inconsistency can be explained by the fact that lysenins were promptly translated into proteins (Figure 10a,b) ameliorating the defense mechanisms of the newly formed body parts.

Interestingly, genes responsible for pro- and anti-inflammatory peptides became overregulated in the early restoration of *Xenopus* larval hindlimb that may be necessary for determining the quality of regeneration [65]. Otherwise in the skin, a plethora of examinations bear out that AMPs participate in modulating the immune response (e.g., cytokine production, cell proliferation, etc.) and enhancing the healing process [60,68]. Similar findings were described among invertebrates (e.g., *D. melanogaster*, *Caenorhabditis elegans*) highlighting that wound healing after tissue injury is conserved progress on an evolutionary scale [68]. Due to the lack of molecular tools targeting earthworm AMPs at the protein level, we can report only our findings concern lysenins; however, other earthworm AMPs need further examination in the regeneration process.

### 2.7. Coelomocyte Depletion Impairs Cell Proliferation and Blastema Regeneration

There is growing evidence that innate immune cells principally myeloid cells are essential to repair mechanisms and their depletion greatly influences the outcome of regeneration [69]. In larvae and adult zebrafish, the association between complete caudal fin regeneration and the crucial role of macrophages was detailed [37]. Other studies underscored the similarities of mammalian and zebrafish macrophages that were able to polarize to M1-like phenotype and alternatively activated M2-like phenotype following tissue injury. This emphasizes that conserved roles of macrophages began to form during the early evolution of lower vertebrates [70,71]. During the axolotl limb regeneration, early depletion of macrophages caused uncontrolled fibroplasia, collagen accumulation, mitigation of surface vasculature and failure in blastema formation [64].

**Figure 11:**
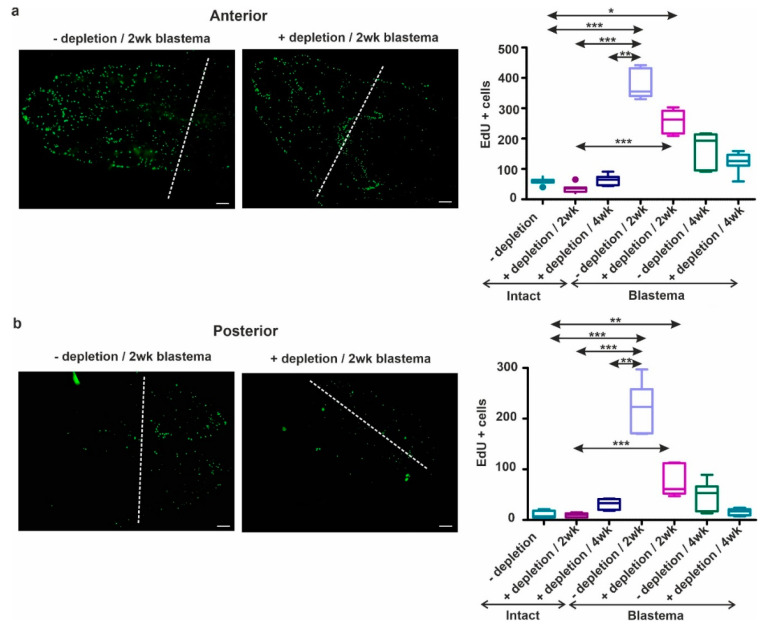
Detection of cell proliferation in the 2-week (**a**) anterior or (**b**) posterior blastema without (left side) or after coelomocyte depletion (right side). Representative images were selected from seven independent experiments of 2-week regenerating earthworms. The level of amputation is indicated by a dashed line. Proliferating cells (green) were enumerated and illustrated on the graphs adjacent to EdU-stained images. Intact earthworms (-) without or (+) with coelomocyte depletion for corresponding intervals used as controls. The amount of proliferating cells (-) without or (+) with coelomocyte ablation, after 2 and 4 weeks, was assessed in the regenerative blastema. Results are depicted on the same graph next to the counted cells of intact segments. The boxes mean interquartile ranges (IQR), whiskers represent lowest and highest values, horizontal lines label median of seven independent (*n* = 7) repetitions. The significance of the data was interpreted by one-way ANOVA with Dunnett’s post-hoc test with GraphPad Prism software (* *p* < 0.05, ** *p* < 0.01, *** *p* < 0.01). Scale bars: 200 μm.

In our study coelomocyte ablation was carried out, that followed by the removal of the anterior or posterior segments. In the absence of coelomocytes, earthworms failed to fully regenerate the lost segments within four weeks. The delayed or malformed restoration was confirmed by morphological observations (Figure S4a,b) and EdU-stainings (Figure 11a,b).

Based on our observations, cell proliferation is more pronounced during anterior (Figure 11a) restoration than the posterior (Figure 11b) counterpart. It also became apparent that the amount of proliferating cells in two-week regenerating animals was much higher in both anterior and posterior regeneration compared to intact and four--week regenerating earthworms. We observed significant differences between intact and two-week (with or without coelomocytes depletion) regenerating earthworms in both anterior (Figure 11a) and posterior (Figure 11b) blastema. We found a significantly higher cell proliferation rate with an approximately two-fold increase in anteriorly regenerating earthworms at two weeks (Figure 11a) compared to the posterior counterpart (Figure 11b). In intact earthworms, we observed no significant differences in the number of dividing cells upon coelomocyte depletion and normal intact earthworms (Figure 11a,b). However, ablation of coelomocytes led to a decreased number of proliferating cells in both two- and four-week anterior or posterior blastema (Figure 11a,b). These results (Figure S4, Figure 11a,b) further strengthen that coelomocyte depletion resulted in a temporary decline of regenerative abilities similar to other model organisms. Although coelomocytes constitute a heterogeneous group with diverse functions, we can surmise that the absence of amoebocytes in the early phase of regeneration contributes to the phenomenon of delayed blastema formation comparable to other species. To strengthen our findings coelomocyte ablation impaired the regeneration of injured cerebral ganglion in earthworms [39].

On the other hand, in the course of coelomocyte ablation coelomic fluid was also depleted, which could accelerate the impaired regeneration process. To this end, both humoral and cellular immune components additively aid the regeneration process. However, the majority of known humoral factors (e.g., lysenin, lumbricin) are produced and secreted from the various subgroups of coelomocytes [15,18]. Future works should mainly focus on the variant and partial roles of each subpopulation in restoration machinery, especially the in vivo ablation of macrophage-like cells to better comprehend their broader roles in tissue homeostasis and repair.

## 3. Materials and Methods

### 3.1. Experimental Set-Up

*E. andrei* earthworms (*Annelida*, *Lumbricidae*) breeding stocks were maintained in the soil at standard laboratory settings [72] and were collected prior to amputations. Earthworms were anesthetized in carbonated water (for 30–60 s) and then the last five anterior- and posterior segments were removed. During the regeneration, period earthworms were maintained in the soil at standard laboratory conditions [72]. One day prior to the removal of the anterior or posterior blastema, earthworms were placed on moist filter paper to empty their intestinal contents. The regenerating blastema was removed at the investigated time points. Tissues were stored in Tissue-Tek O.C.T. cryostat embedding medium (VWR, Radnor, PA) at −80 °C. In all experiments, intact animals with normal anterior or posterior segments were used as controls.

### 3.2. Detection of Cell Proliferation and Programmed Cell Death

To detect cell proliferation, EdU (5-ethynyl-2′-deoxyuridine, 12.5 μg) was injected into the anesthetized earthworms using a Hamilton pipette. In intact animals, the injection was carried out into the 5th segment from the anterior end or the posterior end. For the 2 and 4-week regenerating animals, EdU was injected into the first original (intact) segment adjacent to the regenerative blastema. After 4 h of the EdU injection, the first or last 5 segments were removed from intact animals and the regeneration blastema from injured earthworms. Tissues were stored in a Tissue-Tek O.C.T. cryopreservative embedding medium at −80 °C and sections were prepared as described earlier. Proliferating cells were detected using the Click-iT EdU Imaging Kit (Life Technologies, Carlsbad, CA, USA) according to the manufacturer’s protocol.

In parallel experiments, apoptotic cells were identified throughout the regeneration period using the Click-iT Plus TUNEL Imaging Assay Kit (modified terminal deoxynucleotidyl transferase-dUTP nick end labeling with Alexa Fluor 488 fluorescent dye, Life Technologies) based on the manufacturer’s protocol.

Different controls were prepared for both experiments. In the case of EdU-staining, two types of negative control were exerted: (1) PBS was injected instead of EdU, and (2) tissues from EdU-injected intact animals were incubated by Click-it EdU reaction mix, but without the Alexa Fluor 488 dye. For the TUNEL-labeling: (1) DNase digestion was employed as a positive control, and (2) the Click-it TUNEL reaction mix was used without TdT enzyme (data not shown). Otherwise, the standard protocols were executed. Cell nuclei were counterstained with 4′6-diamino-2 phenylindole dihydrochloride (DAPI, 10 µg/mL, Life Technologies) and sections were covered with a 1:1 mixture of PBS-glycerol [67,73].

### 3.3. Actin Detection

Following EdU-staining the sections were labeled with CF568 Phalloidin conjugates (Invitrogen) to monitor the actin-filament rearrangements [15] in regenerating earthworms. Tissues sections from intact animals were applied as controls.

To visualize alpha-smooth muscle actin (α-SMA), sections were fixed in cold acetone for 10 min, followed by incubation with 5% BSA/PBS for 20 min at room temperature (RT) to avoid non-specific bindings. Mouse anti-human α-SMA monoclonal antibody (mAb) (1:100 in PBS, Dako Agilent, Santa Clara, CA, USA) was applied as the primary antibody for 1 h at RT. After the washing steps, FITC conjugated anti-mouse Ig (BD Biosciences, San José, CA, USA) was used as a secondary antibody. Nuclear staining and section covering were performed as previously detailed. Control slides were also similarly prepared and incubated with mouse IgG1 as a primary antibody.

### 3.4. Immunofluorescence Staining of Coelomocyte Subsets

According to the manufacturer’s instructions of EdU or TUNEL staining, sections were fixed in 4% paraformaldehyde. In this regard, EdU and TUNEL slides were the subjects of immunofluorescence to detect distinct coelomocyte subpopulations [15]. For double labeling, slides were incubated in a blocking solution (EdU: 3% BSA in PBS; TUNEL: 3% BSA + 0.1% in Triton-X/PBS) before adding primary antibodies. Slides were incubated with in house-developed biotin-conjugated anti-lysenin (eleocyte-specific, anti-EFCC5, 1:50) and granular amoebocyte-specific (anti-EFCC4, 1:100) mAbs (mouse IgG; both diluted in blocking solutions) for 1 h at RT [17]. Streptavidin-R-phycoerythrin (1:100; 0.1% in Triton-X/PBS, BD Biosciences) was used as a secondary reagent for 1 h in the dark at RT. For nuclear counterstaining DAPI was applied and subsequently sections were covered with a 1:1 mixture of PBS-glycerol.

### 3.5. Depletion of Coelomocytes

Extrusion of coelomocytes was executed as described previously [74], followed by the amputation of the last five anterior- and posterior segments. Subsequently, animals were replaced in the soil for 2 or 4 weeks. After the regeneration period, the same earthworms were collected again from the soil and the regenerating blastema was removed for common morphological observations (please see in the Supplementary Material, Figure S4). To compare the proliferating cell numbers between depleted and non-depleted earthworms, we applied EdU-injection (please see Section 3.2) after the regeneration period. Coelomocyte depleted intact earthworms used as a negative control.

### 3.6. Image Analysis

Sections were examined using an Olympus BX61 microscope and AnalySIS software (Olympus Hungary, Budapest, Hungary). Cell proliferation data were evaluated from seven independent experiments. Cells were enumerated with ImageJ (NIH) software.

### 3.7. Statistical Analysis

Data analyses were performed by Prism v5.0 (GraphPad Software, La Jolla, CA, USA). The significance of the data was assessed by one-way ANOVA using Dunn’s multiple comparison post hoc test and * *p* < 0.05, ** *p* < 0.01, *** *p* < 0.001 values were considered as statistically significant.

## 4. Conclusions

Regenerative ability is an ancient trait and its success is unambiguously determined by certain factors. So far, scientists are attempting to clarify the perplexing phenomenon of why and how the intensive regeneration capacity has been abolished during evolution [2,8]. The role of the immune system is crucial in the regeneration process and to the best of our knowledge, the regeneration capacity is inversely proportional to the development of the immune system. The obtained recent knowledge provides that regeneration can be considered as an alternative way of embryogenesis based on the molecular signatures. To this end, recent regenerative studies have focused principally on the genes required for development and our understanding of the immunological perspectives in regeneration remains less understood.

Several cellular and molecular alterations of innate immunity were observed in regenerating *E. andrei* earthworms. Coelomocytes contribute to the successful segment renewal, however, immune response-related mRNA expression (*PRR*, *AMPs*) has been attenuated compared to intact animals. This is not unique for earthworms because down-regulation of immune-related genes has been also described in other species during regeneration. This phenomenon may indicate an immature nature of the blastema and the metabolism is also altered compared to differentiated tissues. On the other hand, down-regulation of immune-related genes can be interpreted by distinct allocation strategies and life-history trade-offs between regeneration and immune mechanisms. In this regard, the blastema presumably represents an immunologically tolerated niche that participates in successful regeneration. Our findings provide us further mechanistic insights into the evolutionarily conserved machinery of the restoration. Understanding precisely how innate immune response is involved in tissue repair of invertebrates will indisputably guide us toward a better comprehension of the complex tissue healing in vertebrates.

## Data Availability

The data presented in this study are available in the article.

## Supplementary Materials

The following are available online at https://www.mdpi.com/1422-0067/22/5/2363/s1. Table S1. Lists of primers and GenBank Accession numbers used for qPCR analysis, Figure S1. Detection of α-SMA during anterior (a) and posterior (b) regeneration in intact and 2- and 4 weeks of regenerating earthworms. The level of amputation is indicated by a dashed line. The α-SMA (green) can be seen in intact segments and reorganized fibers of the regenerating blastema. Scale bars: 100 μm (intact), 200 μm (blastema), Figure S2. Detection of apoptosis throughout the anterior regeneration from the early blastema formation (2 and 4 days) to the later phases (2 and 4 weeks) of the restoration period. The proportion of TUNEL positive cells (green) was more pronounced in the early (2–4 days), and in the week 4 blastema. Conversely, the apoptotic activity was remarkably decreased in the 2 wk regenerating segments. Scale bars: 100 µm, Figure S3. Detection of apoptosis throughout the posterior regeneration from the early blastema formation (2 and 4 days) to the later phases (2 and 4 weeks) of the restoration. TUNEL positive cells (green) could be observed during the course of posterior restoration, however, predominantly in the later stages of the blastema (2 and 4 weeks). Scale bars: 100 µm, Figure S4. Morphological observations of the anterior (a–e) and posterior(f–j) regeneration process following depletion of coelomocytes ((a,f): intact; (b,g): 2 days, (c,h): 4 days; (d,i): 2 weeks; (e,j): 4 weeks). H&E staining was performed to observe the impaired tissue regeneration. The level of amputation is indicated by a dashed line. Scale bars: 200 µm.

## Abbreviations

α-SMA	smooth muscle α actin
ACP	Acid phosphatase
ALP	Alkaline phosphatase
AMP	Antimicrobial peptide
ATP	Adenosine triphosphate
A.U.	Arbitrary unit
BSA	Bovine serum albumin
CC	Coelomic cavity
CCF	Coelomic cytolytic factor
DAPI	4′6-diamino-2 phenylindole dihydrochloride
EdU	5-ethynyl-2′-deoxyuridine
EFCC	*Eisenia fetida* coelomocyte clusters
FITC	Fluorescein isothiocyanate
H&E	Hematoxylin-eosin
H_2_O_2_	Hydrogen peroxide
IL	Interleukin
LBSS	*Lumbricus* balanced salt solution
Lumbr	Lumbricin
LuRP	Lumbricin-related peptide
LBP/BPI	Lipopolysaccharide-binding protein/bacterial permeability-increasing protein
NF-κB	Nuclear factor kappa-light-chain-enhancer of activated B cells
PAS	Periodic Acid and Schiff
PRR	Pattern recognition receptor
qPCR	Quantitative polymerase chain reaction
ROS	Reactive oxygen species
SDS-PAGE	Sodium dodecyl sulphate-polyacrylamide gel electrophoresis
SR	Scavenger receptor
TLR	Toll-like receptor
TIR	Toll/interleukin-1 receptor
TUNEL	Terminal deoxynucleotidyl transferase-dUTP nick end labeling
Wnt	Wingless/integrated
wk	week

## References

[1] Bely A.E. (2010). Evolutionary loss of animal regeneration: Pattern and process. Integr. Comp. Biol..

[2] Bely A.E., Nyberg K.G. (2010). Evolution of animal regeneration: Re-emergence of a field. Trends Ecol. Evol..

[3] Mescher A.L., Neff A.W. (2005). Regenerative capacity and the developing immune system. Adv. Biochem. Eng. Biotechnol..

[4] Eming S.A., Krieg T., Davidson J.M. (2007). Inflammation in wound repair: Molecular and cellular mechanisms. J. Investig. Dermatol..

[5] Engelmann P., Bodó K., Najbauer J., Németh P., Cooper E.L. (2018). Annelida: Oligochaetes (Segmented Worms): Earthworm immunity, quo vadis? Advances and new paradigms in the omics era. Advances in Comparative Immunology.

[6] Julier Z., Park A.J., Briquez P.S., Martino M.M. (2017). Promoting tissue regeneration by modulating the immune system. Acta Biomater..

[7] Eming S.A. (2014). Evolution of immune pathways in regeneration and repair: Recent concepts and translational perspectives. Semin. Immunol..

[8] Bely A.E., Sikes J.M. (2010). Latent regeneration abilities persist following recent evolutionary loss in asexual annelids. Proc. Natl. Acad. Sci. USA.

[9] Baranzini N., Pulze L., Acquati F., Grimaldi A. (2020). *Hirudo verbena* as an alternative model to dissect the relationship between innate immunity and regeneration. Invertebr. Surv. J..

[10] Bely A.E. (2006). Distribution of segment regeneration ability in the Annelida. Integr. Comp. Biol..

[11] Varhalmi E., Somogyi I., Kiszler G., Nemeth J., Reglodi D., Lubics A., Kiss P., Tamas A., Pollak E., Molnar L. (2008). Expression of PACAP-like compounds during the caudal regeneration of the earthworm *Eisenia fetida*. J. Mol. Neurosci..

[12] Tessmar-Raible K., Arendt D. (2003). Emerging systems: Between vertebrates and arthropods, the Lophotrochozoa. Curr. Opin. Genet. Dev..

[13] Nyberg K.G., Conte M.A., Kostyun J.L., Forde A., Bely A.E. (2012). Transcriptome characterization via 454 pyrosequencing of the annelid *Pristina leidyi*, an emerging model for studying the evolution of regeneration. BMC Genom..

[14] Cooper E.L., Kauschke E., Cossarizza A. (2002). Digging for innate immunity since Darwin and Metchnikoff. Bioessays.

[15] Engelmann P., Hayashi Y., Bodó K., Ernszt D., Somogyi I., Steib A., Orbán J., Pollák E., Nyitrai M., Németh P. (2016). Phenotypic and functional characterization of earthworm coelomocyte subsets: Linking light scatter-based cell typing and imaging of the sorted populations. Dev. Comp. Immunol..

[16] Engelmann P., Hayashi Y., Bodó K., Molnár L., Ballarin L., Cammarata M. (2016). New aspects of earthworm innate immunity: Novel molecules and old proteins with unexpected functions. Lessons in Immunity: From Single Cell Organisms to Mammals.

[17] Engelmann P., Pálinkás L., Cooper E.L., Németh P. (2005). Monoclonal antibodies identify four distinct annelid leukocyte markers. Dev. Comp. Immunol..

[18] Bodó K., Ernszt D., Németh P., Engelmann P. (2018). Distinct immune- and defense-related molecular fingerprints in separated coelomocyte subsets in *Eisenia andrei* earthworms. Invertebr. Surv. J..

[19] Prochazkova P., Roubalova R., Dvorak J., Navarro Pacheco N.I., Bilej M. (2020). Pattern recognition receptors in annelids. Dev. Comp. Immunol..

[20] Bhambri A., Dhaunta N., Patel S.S., Hardikar M., Bhatt A., Srikakulam N., Shridhar S., Vellarikkal S., Pandey R., Jayarajan R. (2018). Large scale changes in the transcriptome of *Eisenia fetida* during regeneration. PLoS ONE.

[21] He C., Klionsky D.J. (2009). Regulation mechanisms and signaling pathways of autophagy. Annu. Rev. Genet..

[22] Kwong W.H., Tam P.P. (1984). The pattern of alkaline phosphatase activity in the developing mouse spinal cord. J. Embryol. Exp. Morphol..

[23] Szabó R., Ferrier D.E. (2014). The dynamics of alkaline phosphatase activity during operculum regeneration in the polychaete *Pomatoceros lamarckii*. Int. J. Dev. Biol..

[24] Rockey D.C., Weymouth N., Shi Z. (2013). Smooth muscle α actin (Acta2) and myofibroblast function during hepatic wound healing. PLoS ONE.

[25] Darby I.A., Laverdet B., Bonté F., Desmoulière A. (2014). Fibroblasts and myofibroblasts in wound healing. Clin. Cosmet. Investig. Dermatol..

[26] Abnave P., Ghigo E. (2019). Role of the immune system in regeneration and its dynamic interplay with adult stem cells. Semin. Cell Dev. Biol..

[27] Liebmann E. (1943). New light on regeneration of *Eisenia foetida* (SAV). J. Morphol..

[28] Cooper E.L., Roch P. (1984). Earthworm leukocyte interactions during early stages of graft rejection. J. Exp. Zool..

[29] Chaves da Silva P.G., Corrêa C.L., de Carvalho S.L., Allodi S. (2013). The crustacean central nervous system in focus: Subacute neurodegeneration induces a specific innate immune response. PLoS ONE.

[30] Schikorski D., Cuvillier-Hot V., Leippe M., Boidin-Wichlacz C., Slomianny C., Macagno E., Salzet M., Tasiemski A. (2008). Microbial challenge promotes the regenerative process of the injured central nervous system of the medicinal leech by inducing the synthesis of antimicrobial peptides in neurons and microglia. J. Immunol..

[31] Vriz S., Reiter S., Galliot B. (2014). Cell death: A program to regenerate. Curr. Top. Dev. Biol..

[32] Galliot B. (2013). Injury-induced asymmetric cell death as a driving force for head regeneration in Hydra. Dev. Genes Evol..

[33] Hwang J.S., Kobayashi C., Agata K., Ikeo K., Gojobori T. (2004). Detection of apoptosis during planarian regeneration by the expression of apoptosis-related genes and TUNEL assay. Gene.

[34] Holstein T.W., Watanabe H., Ozbek S. (2011). Signaling pathways and axis formation in the lower metazoa. Curr. Top. Dev. Biol..

[35] Gauron C., Rampon C., Bouzaffour M., Ipendey E., Teillon J., Volovitch M., Vriz S. (2013). Sustained production of ROS triggers compensatory proliferation and is required for regeneration to proceed. Sci. Rep..

[36] Willenborg S., Eming S.A. (2014). Macrophages-sensors and effectors coordinating skin damage and repair. J. Dtsch Dermatol. Ges..

[37] Morales R.A., Allende M.L. (2019). Peripheral macrophages promote tissue regeneration in zebrafish by fine-tuning the inflammatory response. Front. Immunol..

[38] Okrzesik J., Kachamakova-Trojanowska N., Jozkowicz A., Morgan A.J., Plytycz B. (2013). Reversible inhibition of reproduction during regeneration of cerebral ganglia and coelomocytes in the earthworm *Dendrobaena veneta*. Invertebr. Surv. J..

[39] Molnar L., Pollak E., Skopek Z., Gutt E., Kruk J., Morgan A.J., Plytycz B. (2015). Immune system participates in brain regeneration and restoration of reproduction in the earthworm *Dendrobaena veneta*. Dev. Comp. Immunol..

[40] Tadokoro R., Sugio M., Kutsuna J., Tochinai S., Takahashi Y. (2006). Early segregation of germ and somatic lineages during gonadal regeneration in the annelid *Enchytraeus japonensis*. Curr. Biol..

[41] Myohara M., Niva C.C., Lee J.M. (2006). Molecular approach to annelid regeneration: cDNA subtraction cloning reveals various novel genes that are upregulated during the large-scale regeneration of the oligochaete, *Enchytraeus japonensis*. Dev. Dyn..

[42] Niva C.C., Lee J.M., Myohara M. (2008). Glutamine synthetase gene expression during the regeneration of the annelid *Enchytraeus japonensis*. Dev. Genes Evol..

[43] Takeo M., Yoshida-Noro C., Tochinai S. (2010). Functional analysis of *grimp*, a novel gene required for mesodermal cell proliferation at an initial stage of regeneration in *Enchytraeus japonensis* (Enchytraeidae, Oligochaete). Int. J. Dev. Biol..

[44] Pfeifer K., Dorresteijn A.W., Fröbius A.C. (2012). Activation of *Hox* genes during caudal regeneration of the polychaete annelid *Platynereis dumerilii*. Dev. Genes Evol..

[45] Pfeifer K., Schaub C., Wolfstetter G., Dorresteijn A. (2013). Identification and characterization of a twist ortholog in the polychaete annelid *Platynereis dumerilii* reveals mesodermal expression of *Pdu-twist*. Dev. Genes Evol..

[46] Huang X.M., Tian Q.N., Bao Z.X., Qin Y.F., Chen S.J., Lu P., Zhang X.L., Zhang Y.Z., Zhang S.T. (2012). Cloning and identification of microRNAs in earthworm (*Eisenia fetida*). Biochem Genet..

[47] Novikova E.L., Bakalenko N.I., Nesterenko A.Y., Kulakova M.A. (2013). Expression of *Hox* genes during regeneration of nereid polychaete *Alitta* (Nereis) *virens* (Annelida, Lophotrochozoa). Evodevo.

[48] Özpolat B.D., Bely A.E. (2015). Gonad establishment during asexual reproduction in the annelid *Pristina leidyi*. Dev. Biol..

[49] Kozin V.V., Kostyuchenko R.P. (2015). *Vasa*, *PL10*, and *Piwi* gene expression during caudal regeneration of the polychaete annelid *Alitta virens*. Dev. Genes Evol..

[50] De Jong D.M., Seaver E.C. (2016). A Stable Thoracic Hox Code and Epimorphosis Characterize Posterior Regeneration in *Capitella teleta*. PLoS ONE.

[51] Škanta F., Procházková P., Roubalová R., Dvořák J., Bilej M. (2016). LBP/BPI homologue in *Eisenia andrei* earthworms. Dev. Comp. Immunol..

[52] Pearson A.M. (1996). Scavenger receptors in innate immunity. Curr. Opin. Immunol..

[53] Brennan J.J., Gilmore T.D. (2018). Evolutionary origins of toll-like receptor signaling. Mol. Biol. Evol..

[54] Škanta F., Roubalová R., Dvořák J., Procházková P., Bilej M. (2013). Molecular cloning and expression of *TLR* in the *Eisenia andrei* earthworm. Dev. Comp. Immunol..

[55] Chen L., DiPietro L.A. (2017). Toll-Like receptor function in acute wounds. Adv. Wound Care.

[56] Ganesan S., Aggarwal K., Paquette N., Silverman N. (2011). NF-κB/Rel proteins and the humoral immune responses of *Drosophila melanogaster*. Curr. Top. Microbiol Immunol..

[57] Canton J., Neculai D., Grinstein S. (2013). Scavenger receptors in homeostasis and immunity. Nat. Rev. Immunol..

[58] Bodó K., Boros Á., Rumpler É., Molnár L., Böröcz K., Németh P., Engelmann P. (2019). Identification of novel lumbricin homologues in *Eisenia andrei* earthworms. Dev. Comp. Immunol..

[59] Opper B., Bognár A., Heidt D., Németh P., Engelmann P. (2013). Revising lysenin expression of earthworm coelomocytes. Dev. Comp. Immunol..

[60] Wilkinson H.N., Hardman M.J. (2020). Wound healing: Cellular mechanisms and pathological outcomes. Open Biol..

[61] Wenger Y., Buzgariu W., Reiter S., Galliot B. (2014). Injury-induced immune responses in *Hydra*. Semin. Immunol..

[62] Peiris T.H., Hoyer K.K., Oviedo N.J. (2014). Innate immune system and tissue regeneration in planarians: An area ripe for exploration. Semin. Immunol..

[63] Vitulo N., Dalla Valle L., Skobo T., Valle G., Alibardi L. (2017). Downregulation of lizard immuno-genes in the regenerating tail and myogenes in the scarring limb suggests that tail regeneration occurs in an immuno-privileged organ. Protoplasma.

[64] Godwin J.W., Pinto A.R., Rosenthal N.A. (2013). Macrophages are required for adult salamander limb regeneration. Proc. Natl. Acad. Sci. USA.

[65] King M.W., Neff A.W., Mescher A.L. (2012). The developing *Xenopus* limb as a model for studies on the balance between inflammation and regeneration. Anat. Rec..

[66] Zattara E.E., Bely A.E. (2013). Investment choices in post-embryonic development: Quantifying interactions among growth, regeneration, and asexual reproduction in the annelid *Pristina leidyi*. J. Exp. Zool. B. Mol. Dev. Evol..

[67] Bodó K., Hayashi Y., Gerencsér G., László Z., Kéri A., Galbács G., Telek E., Mészáros M., Deli A.M., Kokhanyuk B. (2020). Species-specific sensitivity of *Eisenia* earthworms towards nobel metal nanoparticles: A multiparametric in vitro study. Environ. Sci. Nano.

[68] Mangoni M.L., McDermott A.M., Zasloff M. (2016). Antimicrobial peptides and wound healing: Biological and therapeutic considerations. Exp. Dermatol..

[69] Eming S.A., Wynn T.A., Martin P. (2017). Inflammation and metabolism in tissue repair and regeneration. Science.

[70] Nguyen-Chi M., Laplace-Builhe B., Travnickova J., Luz-Crawford P., Tejedor G., Phan Q.T., Duroux-Richard I., Levraud J.P., Kissa K., Lutfalla G. (2015). Identification of polarized macrophage subsets in zebrafish. elife.

[71] Nguyen-Chi M., Laplace-Builhé B., Travnickova J., Luz-Crawford P., Tejedor G., Lutfalla G., Kissa K., Jorgensen C., Djouad F. (2017). TNF signaling and macrophages govern fin regeneration in zebrafish larvae. Cell Death Dis..

[72] Molnár L., Engelmann P., Somogyi I., Mácsik L.L., Pollák E. (2012). Cold-stress induced formation of calcium and phosphorous rich chloragocyte granules (chloragosomes) in the earthworm *Eisenia fetida*. Comp. Biochem. Physiol. A Mol. Integr. Physiol..

[73] Kellermayer Z., Vojkovics D., Dakah T.A., Bodó K., Botz B., Helyes Z., Berta G., Kajtár B., Schippers A., Wagner N. (2019). IL-22-independent protection from colitis in the absence of Nkx2.3 transcription factor in mice. J. Immunol..

[74] Engelmann P., Molnár L., Pálinkás L., Cooper E.L., Németh P. (2004). Earthworm leukocyte populations specifically harbor lysosomal enzymes that may respond to bacterial challenge. Cell Tissue Res..

