# Supplementary material for: Injury-Induced Innate Immune Response During Segment Regeneration of the Earthworm, Eisenia andrei
Source: Int J Mol Sci. 2021 Feb 27;22(5):2363. doi: 10.3390/ijms22052363 (PMC7956685; doi:10.3390/ijms22052363)
Supplement: Supplementary file 1 [file ijms-22-02363-s001.pdf]

SUPPLEMENTARY MATERIAL

Injury-Induced Innate Immune Response During Segment Regeneration of the Earthworm, *Eisenia andrei*

Kornélia Bodó[1], Zoltán Kellermayer[1], Zoltán László[2], Ákos Boros[2], Bohdana Kokhanyuk[1], Péter Németh[1] and Péter Engelmann [1,*]

[1] Department of Immunology and Biotechnology, Clinical Center, Medical School, University of Pécs, Szigeti u, 12, 7643 Pécs, Hungary; bodo.kornelia@pte.hu (K.B.); kellermayer.zoltan@pte.hu (Z.K.); kokhanyukb@gmail.com (B.K.); nemeth.peter@pte.hu (P.N.)

[2] Department of Medical Microbiology and Immunology, Medical School, University of Pécs, Pécs, Hungary; ifj.laszlozoltan@gmail.com (Z.L.); borosakos@gmail.com (Á.B.)

*Correspondence: engelmann.peter@pte.hu (P.E.); +36-72-536-288 (P.E.); +36-72-536-289 (P.E.)

TABLE OF CONTENTS

1. Materials and methods 3
1.1. General histochemistry 3
1.2. Enzyme histochemistry 3
1.3. SDS-polyacrylamide gel electrophoresis and Western blots 3
1.4. RNA-isolation, cDNA synthesis and real-time PCR 3
2. References 5
3. Tables 6
4. Figures 7

1. Materials and methods

1.1. General histochemistry

Cryostat sections (8 μm) were dried overnight and then hematoxylin-eosin (H&E) staining was applied according to the standard procedure. To detect polysaccharides, sections were incubated for 10 min in 1% periodic acid, followed by incubation in Schiff's (PAS) reagent for 10 min as we described earlier [1].

1.2. Enzyme histochemistry

Assessment of acid- and alkaline phosphatase activity was performed as we described previously [1]. For ACP measurements sections were fixed and incubated in the reaction mixture (10 mg of naphthol AS-BI phosphate, Sigma-Aldrich, Budapest, Hungary) dissolved in dimethylformamide (DMF), 4% pararosaniline, 2% aqueous hydrochloric acid, and 2% aqueous sodium nitrite (Sigma-Aldrich) for 3 hours. After the incubation, sections were washed with sodium acetate buffer followed by hematoxylin counterstaining. Monitoring alkaline phosphatase (ALP) was studied with the mixture of nitro blue tetrazolium and 5-bromo-4-chloro-3-indolyl phosphate (dissolved in 70% and 100% DMF, respectively) (Biotium, Fremont, CA) in reaction buffer (10 mM Tris-Base, 100 mM NaCl and 5 mM $MgCl_2$; pH: 9.5) for 20-25 min.

1.3. SDS-polyacrylamide gel electrophoresis and Western blots

Intact ends vs. anterior/posterior blastema (three biological repetitions) were lysed in RIPA buffer (50 mM Tris/HCl, 150 mM NaCl, 1% (v/v) NP-40, 0.5% (w/v) Na-deoxycholate, 5 mM EDTA, 0.1% SDS, pH 8.0) supplemented with protease inhibitor cocktail (Sigma- Aldrich) on ice for 15 min. Lysates were centrifuged at 16 kRCF for 15 min at 4 ºC to separate debris. Protein concentrations of tissue lysates were assessed with BCA Reagent Kit (Pierce, Rockford, IL, USA).

For the SDS-PAGE protein samples were separated on 10% polyacrylamide gels, topped by a 4% stacking gel using the Mini-Protean 3 apparatus (Bio-Rad, Hercules, CA, USA). Proteins were directly transferred onto nitrocellulose membranes in blotting buffer for 2 h at 4 °C. Thereafter, nitrocellulose membranes were incubated in blocking solution (1% BSA/TBS/0.1% Tween-20,) for 1 h at RT. Membranes were incubated with polyclonal rabbit anti-lysenin antibody (1:1500, Pepta Nova GmbH, Sandhausen, Germany) [2] or mouse monoclonal anti-tubulin (1:1000, Sigma-Aldrich) as a loading control in blocking solution overnight at 4 ºC. On the following day, membranes were washed in TBS-T for 30 min and incubated with HRP-conjugated anti-rabbit IgG secondary antibody (1:1000) or anti-mouse IgG secondary antibody (1:1000) in TBS-T for 1 h at RT. Followed the washing steps ECL detection reagent (Super Signal West Pico Plus, Thermo Scientific) was used to visualize the membranes. Chemiluminescent signals were detected by ChemiDoc Imaging System and analyzed by ImageLab software (BioRad).

1.4. RNA-isolation, cDNA synthesis and real-time PCR

Total RNA was extracted from intact and regenerating tissues using NucleoZOL reagent (Macherey-Nagel GmbH, Düren, Germany) and Potter Grinding Chamber (Proscientific, Oxford, CT, USA) according to the manufacturer's instructions. RNA quantity and quality was ascertained with a NanoDrop spectrophotometer. RNA samples were stored at -80 °C. Prior to cDNA preparation, DNase digestion (Amplification Grade DNase I; Sigma-Aldrich) was performed (25

°C for 15 min, 72 °C for 10 min) in order to achieve greater RNA purity. Next, DNase-digested total RNA was transcribed to cDNA using a High Capacity cDNA Reverse Transcription Kit (Thermo Scientific) based on the manufacturer's protocol. Prepared cDNAs were stored at -20 °C and subsequently used as PCR reaction templates [3].

Gene-specific primers [3, 4] were designed by Primer Express Software (Thermo Scientific) (please see Table S1.). Gene expressions were measured by qPCR using a Maxima SYBR Green Master Mix (Thermo Scientific) on an ABI Prism 7500 (Applied Biosystems). The pre-denaturation step of the amplification profile started at 95 °C and lasted for 10 minutes. This was followed by 40 cycles of denaturation (35 s at 95 °C), hybridization (35 s at 58 °C), and elongation (1 min at 72 °C) stages with ultimately a dissociation step. Quantitative measurements were normalized to *RPL17* mRNA level [3]. PCR data were determined from four independent experiments.

2. References

1. Engelmann, P.; Hayashi, Y.; Bodó, K.; Ernszt, D.; Somogyi, I.; Steib, A.; Orbán, J.; Pollák, E.; Nyitrai, M.; Németh, P.; Molnár, L. Phenotypic and functional characterization of earthworm coelomocyte subsets: Linking light scatter-based cell typing and imaging of the sorted populations. *Dev. Comp. Immunol*. **2016** *65*, 41-52. doi: 10.1016/j.dci.2016.06.017.
2. Bodó, K.; Hayashi, Y.; Gerencsér, G.; László, Z.; Kéri, A.; Galbács, G.; Telek, E.; Mészáros, M.; Deli, A.M.; Kokhanyuk, B.; et al. Species-specific sensitivity of Eisenia earthworms towards nobel metal nanoparticles: A multiparametric in vitro study. *Environ. Sci. Nano*. **2020** 7, 3509-3525. doi: 10.1039/c9en01405e.
3. Bodó, K.; Boros, Á.; Rumpler, É.; Molnár, L.; Böröcz, K.; Németh, P.; Engelmann, P. Identification of novel lumbricin homologues in *Eisenia andrei* earthworms. *Dev. Comp. Immunol*. **2019** *90*, 41-46. doi: 10.1016/j.dci.2018.09.001.
4. Bodó, K.; Ernszt, D.; Németh, P.; Engelmann, P. Distinct immune- and defense-related molecular fingerprints in separated coelomocyte subsets in *Eisenia andrei* earthworms. *Invertebr. Surv. J*. **2018** *15*, 338-345. doi: 10.25431/1824-307X/isj.v15i1.338-345.

3. Tables

Table S1. Lists of primers and GenBank Accession numbers used for qPCR analysis

Target gene	Gene Bank accession #	Sequence (5'-3')a	Amplicon size (bp)
RPL 17	BB998250	GCA GAA TTC AAG GGA CTG GA<br>CTC CTT CTC GGA CAG GAT GA	159
TLR	JX898685	ATT GTG TCA AAC GCC TTC GC<br>GTC GGC GAT CTC TTC CAA CA	123
CCF	AF030028	CAT TAA GCC GAC GTT GCT GG<br>CGT CCT GTA GCA TCC GTT GT	145
LBP/BPI	JQ407018	GGT TCG ACC TCC GAC GAT AC<br>GGT CAA CAG GGC GTC CAT TA	107
SR	JX898683	TAG AGG GAC GAC TGG AGG TG<br>CCC ACG AAA CCG TAT CCC AA	115
Lysozyme	DQ339138	GTC GCA TGG ATG TCG GAT CT<br>GCG AGC AGT CCA TCT GAG TT	120
Lysenin	D85846	CTT GTG AGC GAT GTC GGC TA<br>TGA TCC ACA CTG GTG CTT CC	117
Lumbricin	KX816866	ACT CGG AAC GCA AGA ACC AA<br>GGT TCT GCG TGA CCT CCT TC	139
LuRP	KX816867	GGT CGA GAG AAT CAA CCC AAC TA<br>TGC GAG TAC AGG CTC GTT AAC	133

[a]Upper and lower primer sequences indicate forward and reverse primers

4. Figures

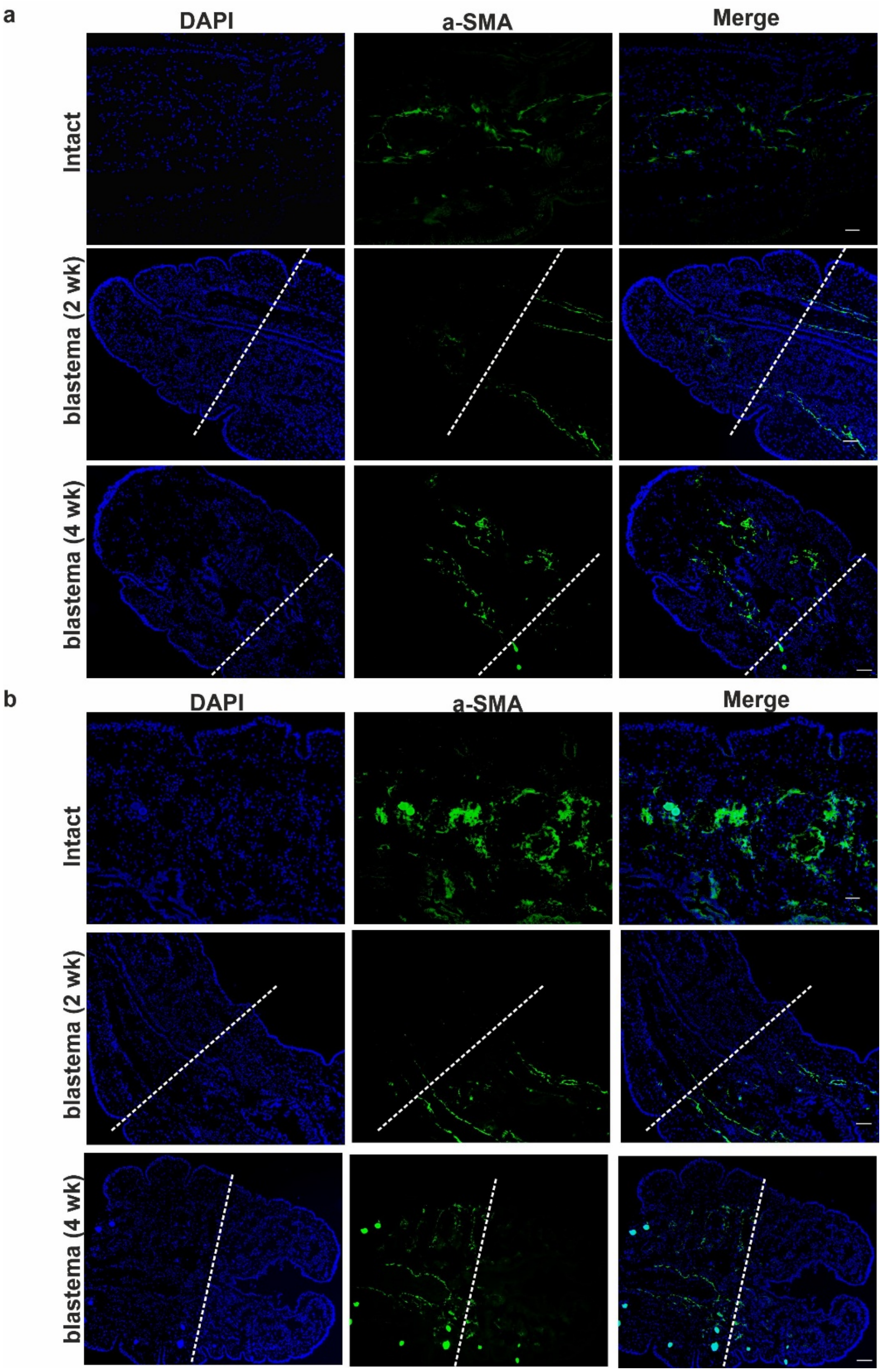

Figure S1. Detection of α-SMA during anterior (**a**) and posterior (**b**) regeneration in intact and 2, - and 4 weeks of regenerating earthworms. The level of amputation is indicated by a dashed line. The α-SMA (green) can be seen in intact segments and reorganized fibers of the regenerating blastema. Scale bars: 100 μm (intact), 200 μm (blastema).

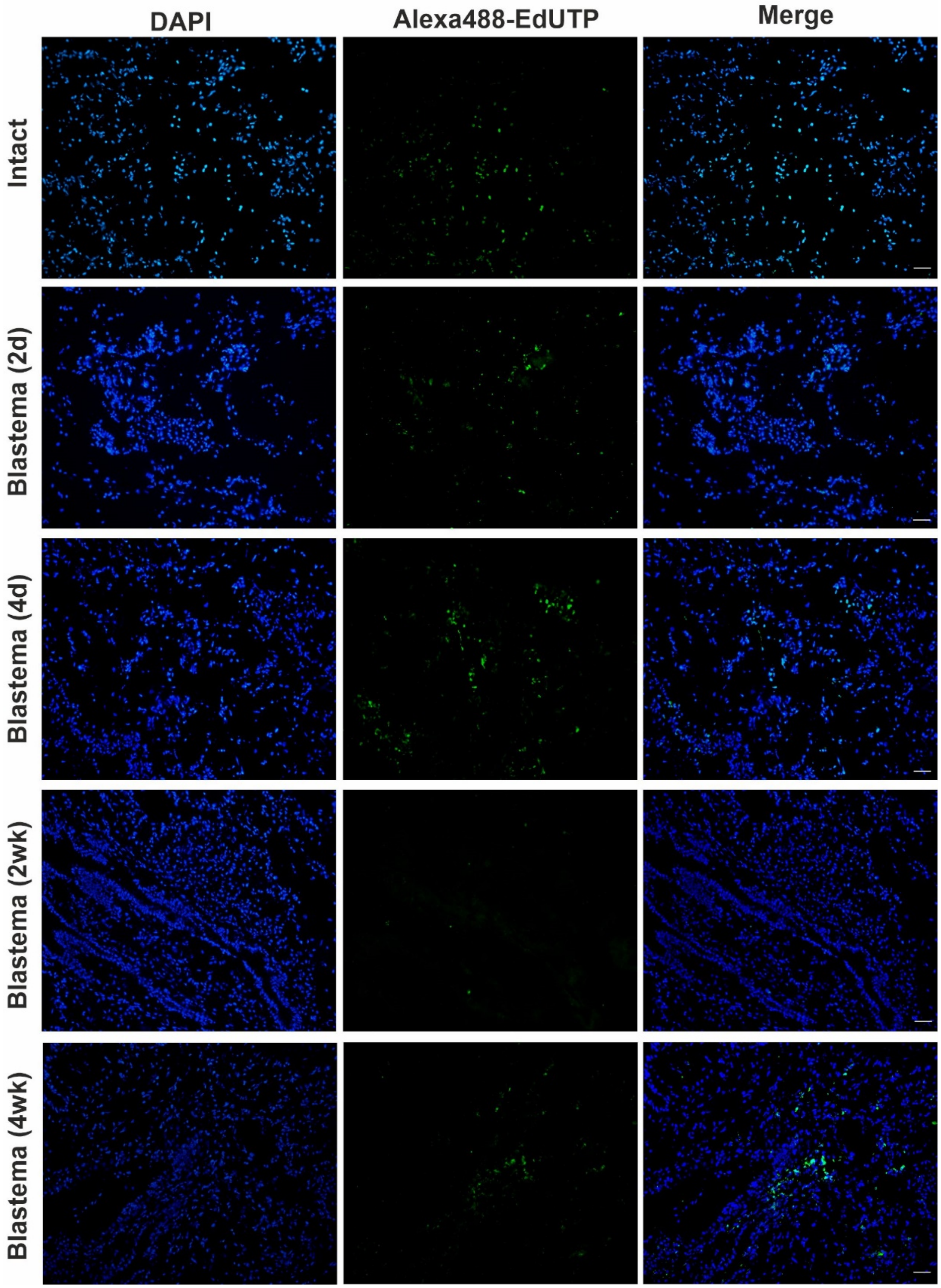

Figure S2. Detection of apoptosis throughout the anterior regeneration from the early blastema formation (2 and 4 days) to the later phases (2 and 4 weeks) of the restoration period. The proportion of TUNEL positive cells (green) was more pronounced in the early (2-4 days), and in the week 4 blastema. Conversely, the apoptotic activity was remarkably decreased in the 2 wk regenerating segments. Scale bars: 100 µm.

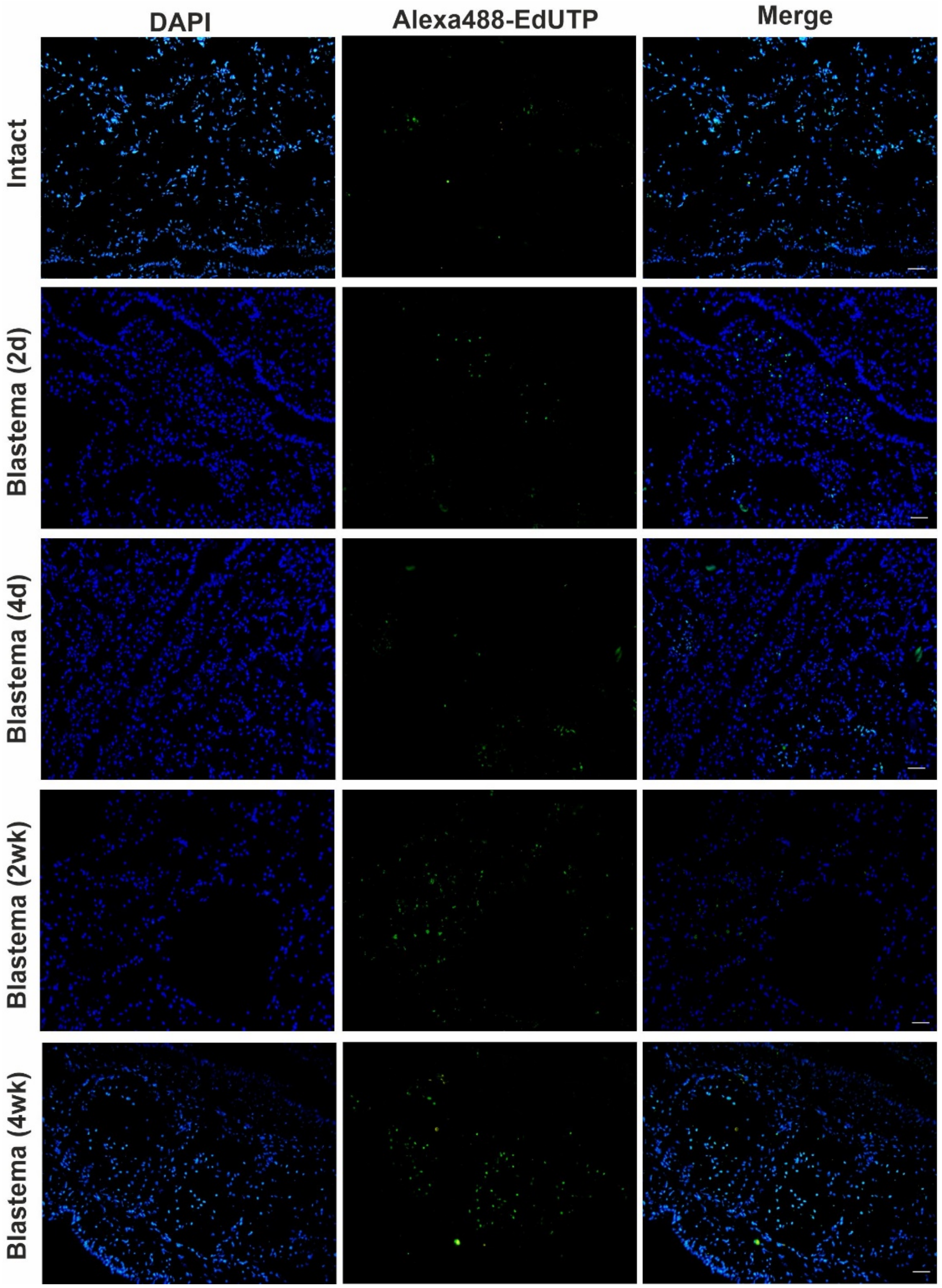

Figure S3. Detection of apoptosis throughout the posterior regeneration from the early blastema formation (2 and 4 days) to the later phases (2 and 4 weeks) of the restoration. TUNEL positive cells (green) could be observed during the course of posterior restoration, however, predominantly in the later stages of the blastema (2 and 4 weeks). Scale bars: 100 µm.

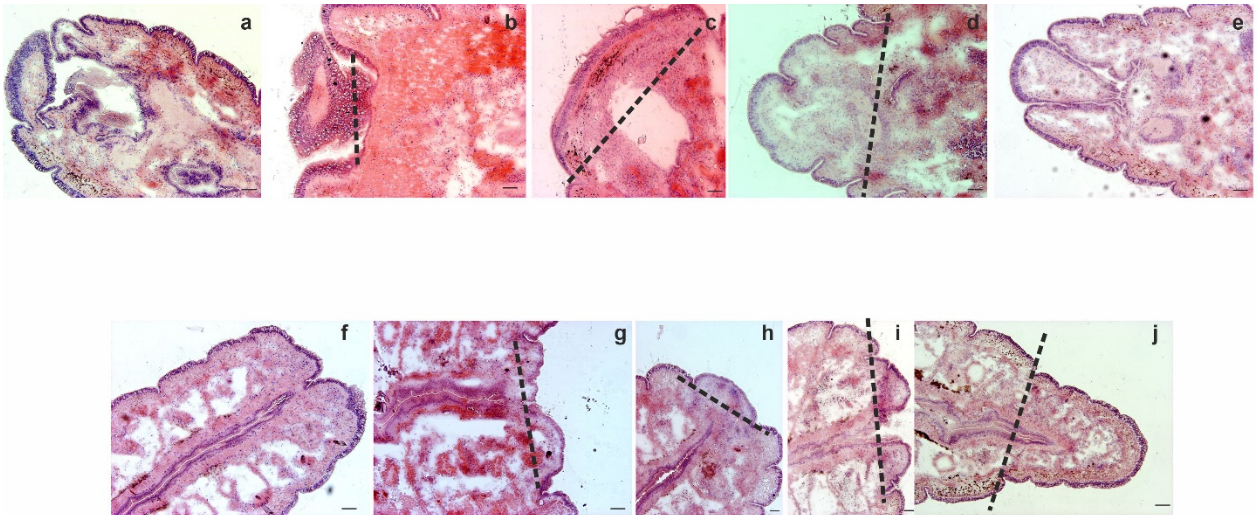

Figure S4. Morphological observations of the anterior (**a-e**) and posterior(**f-j**) regeneration process following depletion of coelomocytes (**a**, **f**: intact; **b**, **g**: 2 days, **c**, **h**: 4 days; **d**, **i**: 2 weeks; **e, j**: 4 weeks). H&E staining was performed to observe the impaired tissue regeneration. The level of amputation is indicated by a dashed line. Scale bars: 200 μm.
